# Fatty acid synthase overexpression: target for therapy and reversal of chemoresistance in ovarian cancer

**DOI:** 10.1186/s12967-015-0511-3

**Published:** 2015-05-07

**Authors:** Dirk O Bauerschlag, Nicolai Maass, Peter Leonhardt, Frederik A Verburg, Ulrich Pecks, Felix Zeppernick, Agnieszka Morgenroth, Felix M Mottaghy, Rene Tolba, Ivo Meinhold-Heerlein, Karen Bräutigam

**Affiliations:** Department of Gynecology and Obstetrics, University Medical Center RWTH Aachen, Pauwelsstrasse 30, 52074 Aachen, Germany; Department of Nuclear Medicine, University Medical Center RWTH Aachen, Pauwelsstrasse 30, 52074 Aachen, Germany; Institute for Laboratory Animal Science, University Medical Center RWTH Aachen, Pauwelsstrasse 30, 52074 Aachen, Germany

**Keywords:** Ovarian cancer, FASN, FASN inhibitor, Cerulenin, Biomolecular marker, Cisplatin, Resistance, Chemotherapy, ^18^ F-fluoromethylcholine, Imaging

## Abstract

**Background:**

Fatty acid synthase (FASN) is crucial to *de novo* long-chain fatty acid synthesis, needed to meet cancer cells’ increased demands for membrane, energy, and protein production.

**Methods:**

We investigated FASN overexpression as a therapeutic and chemosensitization target in ovarian cancer tissue, cell lines, and primary cell cultures. FASN expression at mRNA and protein levels was determined by quantitative real-time polymerase chain reaction and immunoblotting and immunohistochemistry, respectively. FASN inhibition’s impact on cell viability, apoptosis, and fatty acid metabolism was assessed by 3-(4,5-dimethylthiazol-2-yl)-2,5-diphenyltetrazolium-bromide assay, cell death detection enzyme-linked immunosorbent assay, immunoblotting, and ^18^ F-fluoromethylcholine uptake measurement, respectively.

**Results:**

Relative to that in healthy fallopian tube tissue, tumor tissues had 1.8-fold average FASN protein overexpression; cell lines and primary cultures had 11-fold–100-fold mRNA and protein overexpression. In most samples, the FASN inhibitor cerulenin markedly decreased FASN expression and cell viability and induced apoptosis. Unlike concomitant administration, sequential cerulenin/cisplatin treatment reduced cisplatin’s half maximal inhibitory concentration profoundly (up to 54%) in a cisplatin-resistant cell line, suggesting platinum (re)sensitization. Cisplatin-resistant cells displayed lower ^18^ F-fluoro-methylcholine uptake than did cisplatin-sensitive cells, suggesting that metabolic imaging might help guide therapy.

**Conclusions:**

FASN inhibition induced apoptosis in chemosensitive and platinum-resistant ovarian cancer cells and may reverse cisplatin resistance.

**Electronic supplementary material:**

The online version of this article (doi:10.1186/s12967-015-0511-3) contains supplementary material, which is available to authorized users.

## Background

State-of-the-art ovarian carcinoma treatment involves cytoreductive surgery followed by first-line platinum-based and taxane-based chemotherapy [1,2]. In half of cases, however, relapse occurs within 2 years and resistance against platinum-based agents rapidly develops, resulting in a 5-year survival rate of only 30% in patients diagnosed with advanced disease, i.e., 70% of all patients with the neoplasm [2,3]. Thus a strong need exists to identify novel therapeutic strategies.

Overexpression of the multi-enzyme protein fatty acid synthase (FASN) potentially could serve as the target of one such strategy. FASN is one of the key enzymes in *de novo* long-chain fatty acid synthesis. Cancer cells rely upon this process to meet their markedly increased demands for membrane and energy production and protein synthesis [4,5].

Three main factors provide rationale for investigating FASN overexpression in ovarian carcinoma. First, there is evidence of this phenomenon in this tumor. In one study [6], immunohistochemical analysis showed elevated synthesis of the protein in >75% of ovarian carcinoma samples. Additionally, in a correlation analysis of differentially-expressed seeding genes identified by a >12,500-gene oligonucleotide microarray [7], we found FASN to be overexpressed in serous papillary ovarian cancer samples versus normal ovarian surface epithelium. FASN overexpression was especially prominent in G2 and G3, i.e., high-grade, serous tumors, which have particularly poor outcomes [7-9]. Indeed, elevated FASN expression has been linked to negative prognosis and reduced disease-free survival in many other neoplasms [10,11].

Second, FASN overexpression has been described in tumor cell lines in which chemotherapy resistance was induced by culture in drug-containing media. Two-fold to three-fold increased FASN promoter activity was demonstrated in breast cancer cells incubated in etoposide-containing media compared to those cultured in drug-free media, although no such effect was observed following cisplatin incubation [12]. Elsewhere, stronger FASN expression was described in a paclitaxel-resistant hepatocellular carcinoma cell line, Hep3B, than in its paclitaxel-sensitive parental clone [13].

Third, single-agent administration of a FASN inhibitor, C93, blocked growth of carboplatin-resistant, and, especially, paclitaxel-resistant ovarian cancer cell lines [14]. However, effects on tumor cell growth of combining a FASN inhibitor and a chemotherapeutic drug were not investigated.

We thus hypothesized that specific FASN inhibition could exert therapeutic effects in highly FASN-expressing ovarian cancer cells, including re-inducing chemosensitivity in platinum-resistant cells. Therefore, we conducted the present study to confirm FASN overexpression and to investigate the effects of two specific FASN inhibitors in ovarian cancer cells, including platinum-resistant cells.

## Methods

### Overview

We performed three sets of experiments. In the first set, we sought to confirm earlier findings of FASN overexpression. We therefore immunohistochemically analyzed an ovarian cancer tissue microarray (TMA). Additionally, we used quantitative real-time polymerase chain reaction (qRT-PCR) and Western Blot (WB), respectively, to analyze FASN overexpression at mRNA and protein levels in 3 established ovarian cancer cell lines and 1 additional cell line in which we induced cisplatin resistance. Further, since *ex vivo* cultures much more closely approximate tumor behaviour than do cell lines, we performed the qRT-PCR and WB studies in primary cell cultures derived from fresh ovarian cancer material from 3 patients. In these experiments, healthy fallopian tube tissue was used as a control [15].

In the second set of experiments, we preclinically evaluated FASN inhibition as a therapeutic strategy in ovarian carcinoma. Specifically, we assessed the effects on FASN, AKT, and ERK protein expression, cell viability, and apoptosis (reflected by mononucleosomes and oligonucleosomes and PARP cleavage) of two FASN inhibitors as single agents, or one of those agents combined with cisplatin. Experiments were performed in the same tumor cell lines and in primary cultures of tumor tissue (n = 3: one G2 and two G3) and healthy fallopian tube tissue (n = 1). To prove FASN inhibitor specificity, we reversed the effect on protein expression of the pro-proliferative kinases AKT and ERK, cell viability, and apoptosis by supplementation with palmitic acid (PA), the final product of FASN reaction.

In the third set of experiments, we assessed metabolic activity changes induced by FASN inhibition alone or combined with cisplatin administration in a cisplatin-resistant cell line versus its parental cisplatin-sensitive cell line. One analogue of a FASN metabolite, ^18^ F-fluoromethylcholine (^18^ F-FCH), and ^18^ F-2-fluorodeoxyglucose (^18^ F-FDG), a glucose analogue providing a marker of tissue metabolism, were used to evaluate effects on fatty acid metabolism and glycolysis, respectively.

Experiments were performed, always in triplicate, either once (cell death detection enzyme-linked immunosorbent assay [CDDE]), twice (^18^ F-FCH uptake), or three times (all others). We report the average of all iterations of each experiment.

### Human biospecimens and ethics

#### TMA

As previously described in depth [16], a TMA was constructed using 8% formalin-fixed, paraffin-embedded tumor. The material was contributed by 104 patients with pathologist-confirmed low malignant potential (LMP) (n = 6), G1 (n = 9), G2 (n = 42), or G3 (n = 47) epithelial ovarian cancer of mostly serous papillary histology treated at the University of Freiburg Department of Gynecology and Obstetrics, Freiburg, Germany, from 1993–1998. Institutional review board approval was granted and informed consent obtained before tissue collection. Material of 12 healthy controls were provided by the Rheinisch-Westfälische Technische Hochschule (RWTH) Centralized Biomaterial Bank (cBMB) according to their regulations, after RWTH Aachen Medical Faculty Ethics Committee approval (decision EK 206/09).

#### Ovarian cancer cell lines

Three established ovarian cancer cell lines (provided by I. M.-H.) possessing three different levels of resistance (sensitive, intermediate and resistant, respectively) to cytotoxic agents were utilized: Hey, Igrov-1 and Skov-3. Additionally, using Hey, the most chemotherapy-sensitive of these lines [17] as the parent, we developed a fourth, cisplatin-resistant line (Hey_cis_), as previously described in detail [18]. All cell lines were cultivated in Roswell Park Memorial Institute (RPMI) 1640 medium containing 10% fetal bovine serum, 1% penicillin, and 1% streptomycin, and, in the case of Hey_cis_, 6,67 μmol/L cisplatin.

#### Primary cell culture material

Ovarian tumor and fallopian tube samples (n = 3 and 1 patients, respectively) were provided by the Rheinisch-Westfälische Technische Hochschule (RWTH) Centralized Biomaterial Bank (cBMB) according to their regulations, after RWTH Aachen Medical Faculty Ethics Committee approval (decision EK 206/09). After collection, tissue was placed on ice and minced using a scalpel in a cell culture dish with RPMI medium and collagenase. This suspension was incubated under gentle agitation at 37°C in a hybridization oven for ≥2 h, then centrifuged at 1700 rpms for 5 min. The cell pellet was transferred within medium into culture flasks for incubation. After splitting at least once, cells were used for experiments.

#### *FASN inhibit*ors

We selected cerulenin and C75 for study based on preclinical data showing cytotoxic effects in a number of tumors, including limited data in ovarian cancer [12,19-21]. Both drugs also were chosen for their commercially availability, published chemical structures (unlike the case with e.g. C93), and suitability for cell culture experiments.

##### Cerulenin

A natural antimycotic isolated from *Cephalosporium caerulens*, cerulenin contains an epoxy group that reacts with FASN’s ketoacyl synthase domain [22]. Cerulenin was among the first compounds found to inhibit FASN, and thereby to induce apoptosis in breast cancer cell lines and the drug delayed disease progression in an ovarian cancer xenograft model [23,24].

##### C75

A cerulenin-derived, semi-synthetic FASN inhibitor lacking cerulenin’s reactive epoxy group [4], C75 is more chemically stable than is its parent. C75 showed significant antitumor effects in human cancer cell lines [25], and ovarian cancer [26], breast cancer [25], and prostate cancer [27] xenografts.

To confirm the specificity of FASN inhibitor, we co-incubated the cells with PA.

### Experimental methods

Details on immunohistochemical analysis, cell culture, RNA isolation, reverse transcription, qRT-PCR, WB, 3-(4,5-dimethylthiazol-2-yl)-2,5-diphenyltetrazolium-bromide (MTT) assay, and CDDE assay methodology are provided in the Supplementary Materials (Additional file 1).

#### FASN inhibitor and cisplatin treatment

For FASN RNA isolation and protein expression experiments, 4x10^5^ cells were seeded in 6-well plates. For cell viability assay experiments, 5x10^3^ cells were seeded in 96-well plates. For FASN protein expression and cell viability assay experiments, the cells were left untreated or were incubated with 35 μmol/L cerulenin or 35 μmol/L C75. FASN inhibitor treatment lasted 72 h in ovarian cancer cell lines and, because of the slower growth of primary cell culture, was extended to 96 h in that setting. For investigating apoptosis induction by CDDE assay, 5x10^3^ cells were seeded in 96-well plates and were left untreated or treated with 25 μmol/L cerulenin for 24 h. For experiments exploring the potential reversal of cisplatin resistance, cells were treated with cerulenin and cisplatin simultaneously as well as sequentially. For simultaneous administration assessments, various cisplatin doses ranging from 0.31–10 μmol/L (Hey) or 0.31–40 μmol/L (Hey_cis_) were combined with 8.75 μmol/L or 17.5 μmol/L cerulenin, or as a comparator, were applied as monotherapy; incubation time was 72 h in all cases. For sequential application assessments, cells were preincubated for 6 h with media containing 8.75 μmol/L or 17.5 μmol/L cerulenin, the medium was completely removed and the cells were incubated an additional 72 h with various cisplatin doses ranging from 0.31–10 μmol/L (Hey) or 0.31–40 μmol/L (Hey_cis_). Alternatively, the cells were given only the cisplatin treatment (comparator). To prove FASN inhibitor effect on protein expression of AKT, ERK, FASN, and PARP, cells were preincubated for 6 h with media containing 17.5 μmol/L cerulenin, the medium was completely removed, and the cells were incubated an additional 72 h with 1.6 μmol/L cisplatin (Hey; half maximum inhibitory concentration (IC50) of cisplatin in Hey cells treated with 17.5 μmol/L cer) and 6.6 μmol/L cisplatin (Hey_cis_; IC50 of cisplatin in Hey_cis_ treated with 17.5 μmol/L cer). Alternatively, the cells were given either cisplatin or cerulenin treatment or left untreated (comparator). For reversal of the effect of FASN inhibitor, previous preparation was repeated and additionally, the samples were preincubated with 50 μmol/L PA.

#### ^18^ F-FCH/^18^ F-FDG uptake

The ^18^ F-FCH and ^18^ F-FDG cellular uptake experiments were performed in Hey or Hey_cis_, the platinum-sensitive parent/platinum-resistant daughter cell lines available to us. For ^18^ F-FCH uptake measurements, 4×10^5^ cells were seeded in 6-well plates. Cerulenin was used alone in 8.75 μmol/L or 17.5 μmol/L doses. Cisplatin was applied as a single agent or in combination with cerulenin in 0.63 μmol/L, 1.25 μmol/L, 2.5 μmol/L, or 5 μmol/L doses (Hey) or 1.25 μmol/L, 2.5 μmol/L, 5 μmol/L, or 10 μmol/L doses (Hey_cis_). Drug combinations were administered with cerulenin given 6 h before cisplatin, afterwards the medium completely removed and the cells incubated an additional 72 h. Next, the cells were incubated with 0.5 MBq tracer/mL per well for 4 h. Thereafter, the cells were washed with phosphate-buffered saline (PBS) and the intracellular accumulated radioactivity was quantified using a gamma counter (Wizard 2480; PerkinElmer Life and Analytical Sciences, Downers Grove, IL, USA). The post-incubation measured radioactivity was divided by the administered radioactivity and normalized to protein content.

#### Statistics

Analyses for immunhistochemistry experiments were done using SAS 9.2 (SAS Institute Inc., Cary, NC, USA). Non-parametric testing for intergroup differences was performed using Kruskal-Wallis and Mann–Whitney *U* tests. All other experiments were analyzed using Student’s *t*-test. *P* < 0.05 was considered statistically significant.

## Results

### FASN expression

#### TMA

**Figure 1:**
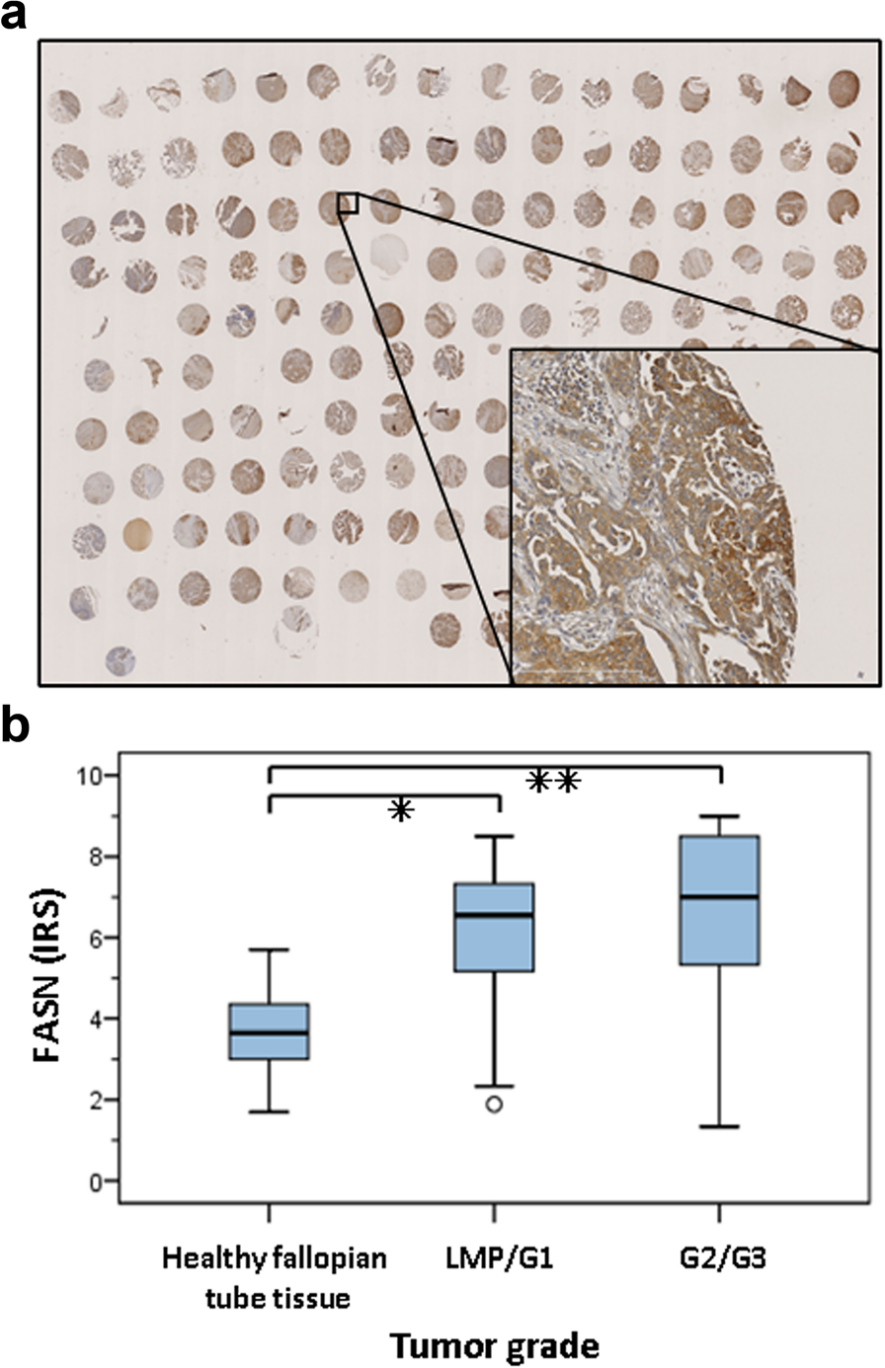
Immunhistochemical analyses of fasn protein expression in patient material. Immunhistochemical analyses of FASN protein expression in a TMA comprising formalin-fixed, paraffin-embedded samples of ovarian cancers of different grades (6 LMP, 9 G1, 42 G2, and 47 G3 tumors) and histological subtypes (serous papillary, mucinous, or endometrioid) from 104 patients versus in 12 healthy fallopian tissue samples. **(a)** Representative TMA slide immunohistochemically-stained with FASN antibody showed strong FASN expression in ovarian cancer. **(b)** Statistical evaluation of FASN expression applying the immunoreactive score (IRS), which incorporates protein staining intensity and the percentage of protein-positive cells. Statistically significant FASN overexpression was proven for LMP/G1 tumors or G2/G3 tumors vs. normal tissues (respectively *P < 0.005 and **P < 0.001, *U* test).

As illustrated in Figure 1, strong FASN overexpression was detected in the majority of tumor samples compared to healthy fallopian tube tissue samples. Relative to that in fallopian tube tissue, FASN protein expression – by IRS = immunoreactive scoring - was on average elevated 1.6-fold in LMP/G1 tumor samples and 1.8-fold in G2/G3 tumor samples. The differences with healthy tissue were statistically highly significant (respectively, *P* = 0.004 and *P* < 0.001, *U* test), but FASN expression did not differ between G2/G3 versus LMP/G1 tumor samples (*P* = 0.169, *U* test).

#### Ovarian cancer cell lines

**Figure 2:**
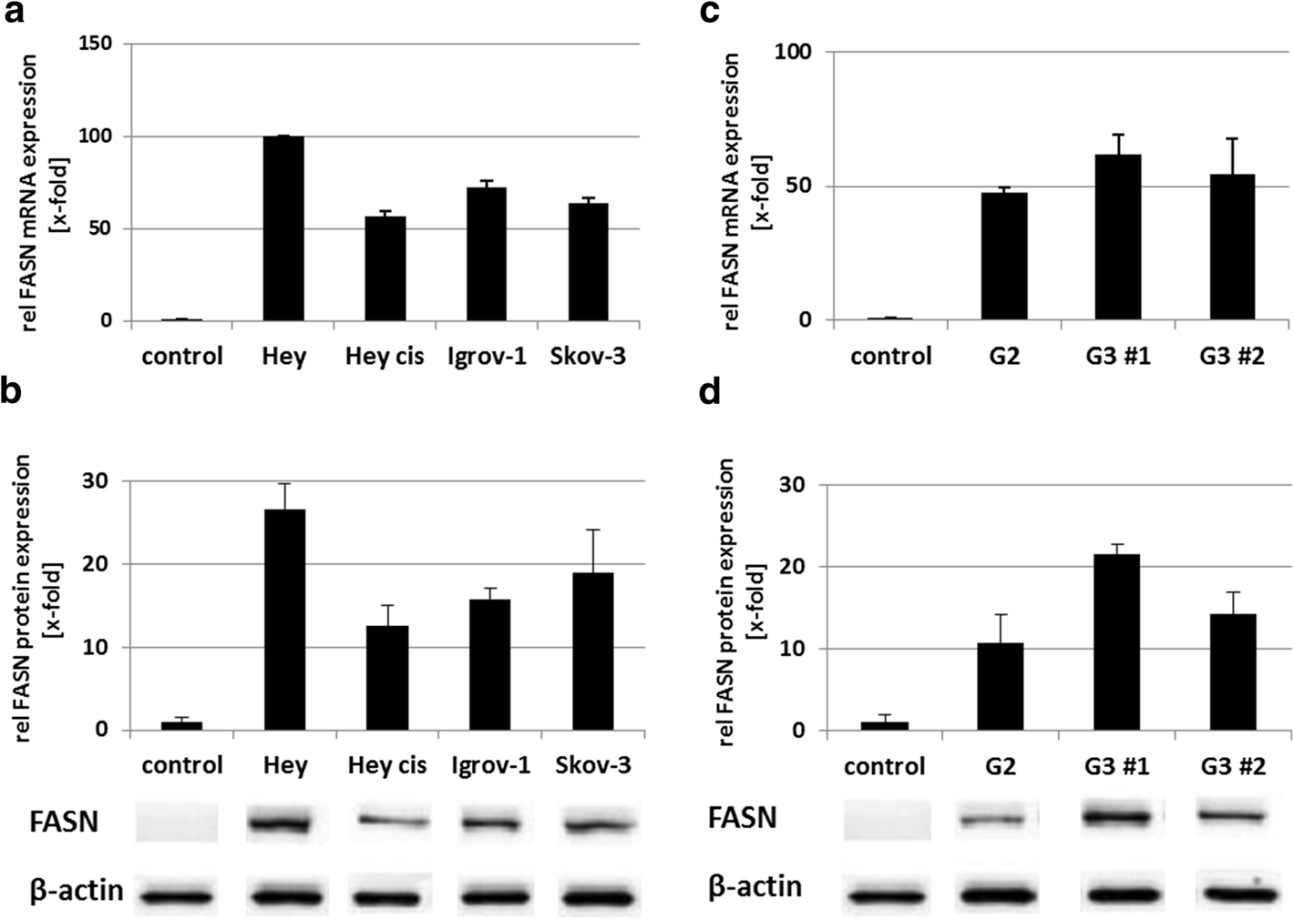
FASN mRNA and protein expression in cell lines and *ex vivo* tumor material. FASN mRNA and protein expression in the (cisplatin-resistant*) ovarian cancer cell lines, Hey, Hey_cis_*, Igrov-1 and Skov-3 and in *ex vivo* tumor material relative (rel) to that in healthy fallopian tube tissue (control). **(a)** By qRT–PCR, all cell lines displayed FASN mRNA overexpression ranging from 56-fold to 100-fold. **(b)** By WB, FASN protein expression was enhanced in all cell lines (12-fold–27-fold). Representative immunoblots for FASN and β-actin (control) of each respective cell line are depicted below the graph. **(c)** By qRT-PCR, FASN mRNA expression was analyzed in primary cultures derived from moderately-differentiated and poorly-differentiated ovarian cancers (G2, G3 #1, and G3 #2) and healthy fallopian tube tissue (control). The cancer cells showed a median (min.–max.) 54-fold (47-fold–62-fold) FASN mRNA overexpression. **(d)** By WB, FASN protein expression was 11-fold– 22-fold stronger in the primary ovarian cancer cell cultures G2, G3 #1, and G3 #2. Representative immunoblots for FASN and β-actin (control) of each respective primary culture are depicted below the graph.

FASN mRNA expression was 100-fold higher in Hey, 72-fold higher in Igrov-1, 63-fold higher in Skov-3, and 56-fold higher in Hey_cis_ than in healthy fallopian tube tissue (Figure 2a). Protein expression analyses confirmed post-transcriptional FASN overexpression (12-fold to 27-fold) in these cell lines, most markedly in the Hey cell line, which was proven to be multidrug-sensitive [18] (Figure 2b).

#### Primary ovarian cancer cell culture

FASN also was highly overexpressed at both mRNA and protein levels in primary culture of high-grade ovarian carcinoma cells, although to a lesser extent than in ovarian cancer cell lines. The single G2 culture and two G3 cultures studied showed a median (min.–max.) 54-fold (47-fold–62-fold) stronger FASN mRNA expression (Figure 2c) and a median (min.–max.) 16-fold (11-fold–22-fold) stronger FASN protein expression (Figure 2d) than that in healthy fallopian tube tissue.

### FASN inhibition

#### Cerulenin vs. C75

**Figure 3:**
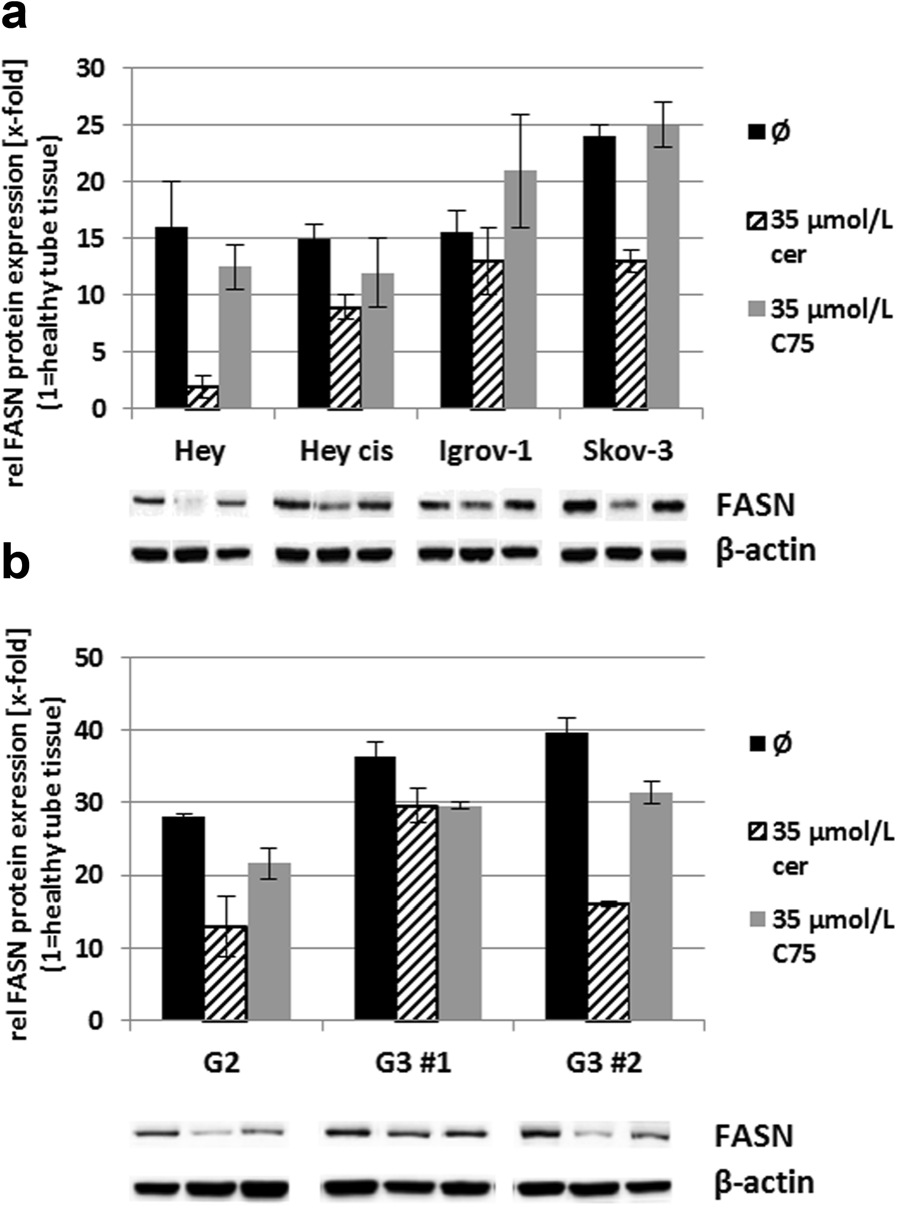
Effect of the FASN inhibitors cerulenin and C75 on FASN expression. Effect of the FASN inhibitors cerulenin and C75 on FASN expression of ovarian cancer cell lines and *ex vivo* tumor material. **(a)** Immunoblot and quantitative analyses of relative (rel) FASN protein expression of the four selected ovarian cancer cell lines Hey, Hey_cis_, Igrov-1, and Skov-3 when untreated (Ø) or after treatment with cerulenin (cer) or C75. Treatment with 35 μmol/L cerulenin typically markedly reduced FASN protein expression, whereas treatment with 35 μmol/L C75 caused just a slight diminution or no diminution of such expression in the investigated cell lines. **(b)** Immunoblot and quantitative analyses of relative (rel) FASN protein expression of primary ovarian cancer cell cultures G2, G3 #1, and G3 #2 when untreated (Ø) or after treatment with cerulenin (cer) or C75. As in the cell lines, in two of three cases the inhibitory effect of cerulenin exceeded that of C75.

**Figure 4:**
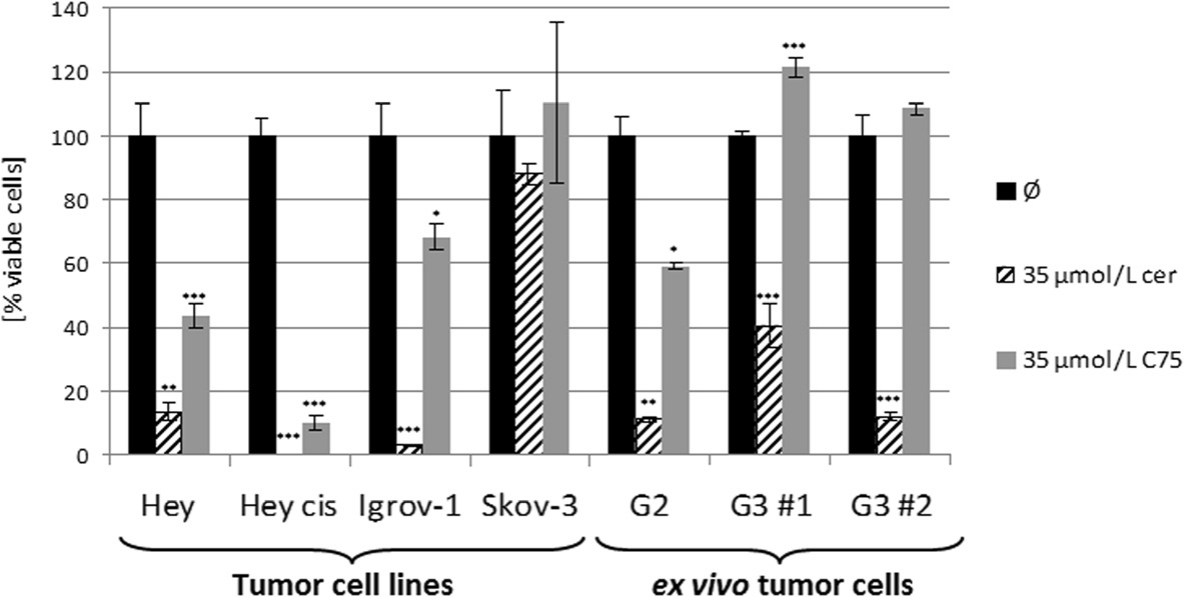
Effect of the FASN inhibitors cerulenin and C75 on cell viability. Effect of the FASN inhibitors cerulenin and C75 on cell viability of ovarian cancer cell lines and *ex vivo* tumor material. The four selected ovarian cancer cell lines, Hey, Hey_cis_, Igrov-1, and Skov-3, and primary ovarian cancer cell cultures G2, G3 #1, and G3 #2 were treated with either 35 μmol/L cerulenin or 35 μmol/L C75. Cell viability was investigated by the MTT assay. Except in the multidrug-resistant cell line Skov-3, the growth of which was hardly affected by either FASN inhibitor, cerulenin (cer) achieved a stronger reduction of cell viability, ≥90% in the other illustrated ovarian cancer cell lines and in two of three primary cancer cell cultures. By contrast, C75 caused <50% inhibition in the Hey and Igrov-1 cell lines and the G2 cell culture and no inhibition in the G3 cell cultures, relative to untreated cells/controls (Ø) (***P < 0.001; **P < 0.01; *P < 0.05).

In ovarian cancer cell lines and primary cultures cerulenin, decreased FASN protein expression (Figure 3), *de novo* fatty acid synthesis, cell growth, and cell viability much more effectively than did C75. Incubation with 35 μmol/L of either FASN inhibitor alone for 72 h substantially inhibited cell viability in all studied ovarian cancer cell lines. However, the strongest effect on cell viability with >90% inhibition was achieved by cerulenin in almost all lines, except the multidrug-resistant Skov-3, in which cerulenin reduced cell viability by just 12%. In comparison, C75 caused only up to 50% inhibition (Figure 4). Cerulenin at a concentration of 8.75 μmol/L reduced Hey_cis_ cell viability by 30%.

In primary cultures, cerulenin diminished cell viability by 60%–90%, whilst C75 decreased cell viability by 40% in G2 culture but had no apparent effect on G3 cell viability (Figure 4).

**Figure 5:**
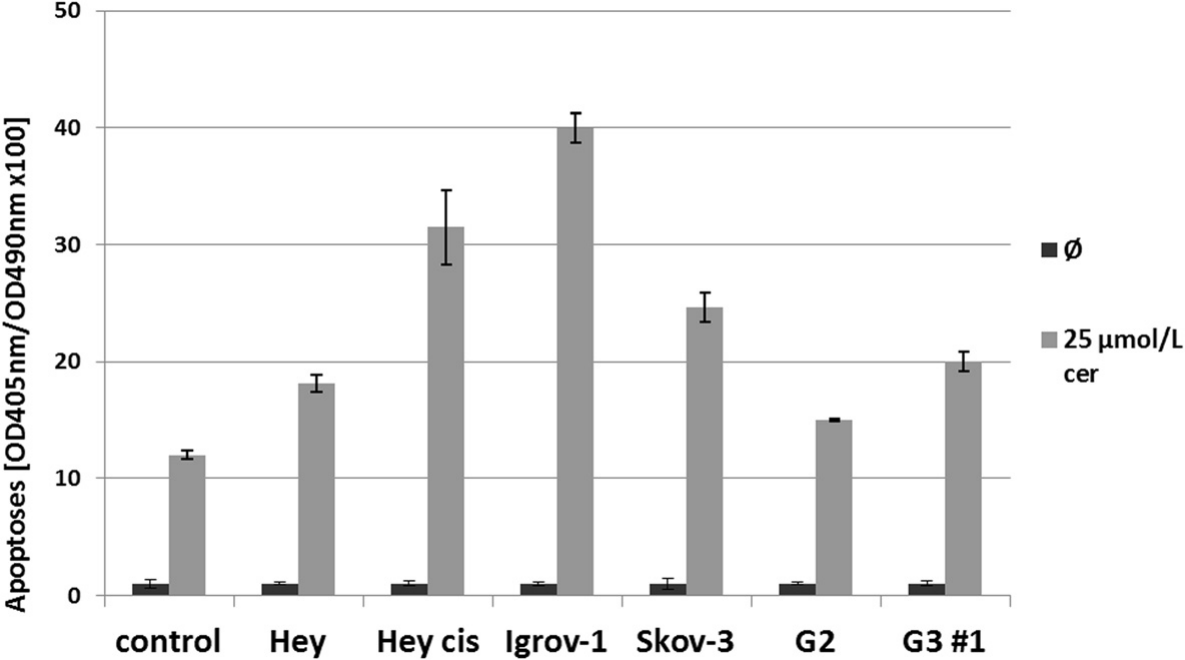
Effect of the FASN inhibitor cerulenin on apoptosis. Effect of cerulenin on apoptosis in ovarian cancer cell lines and *ex vivo* tumor material. CDDE was used to analyze programmed cell death in the four selected ovarian cancer cell lines, Hey, Hey_cis_, Igrov-1, and Skov-3, and two primary ovarian cancer cell cultures G2 and G3 #1, after administration of 25 μmol/L cerulenin. Caspase activation was increased from 1.5- to 3.3-fold in the ovarian cancer cell lines and from 1.3- to 1.7-fold in the *ex vivo* cancer cells relative to that (caspase activation induced by cerulenin to 12x100) in healthy tube tissue cells (controls; Ø). Optical density (OD) = 405 nm (490-nm reference wavelength).

A cerulenin concentration as low as 25 μmol/L applied for 24 h showed profound apoptosis induction with the strongest effect in Igrov-1. CDDE detected from 1.5- to 3.3-fold increased apoptosis induction in ovarian cancer cell lines and at least from 1.3- to 1.7-fold increased caspase activation in primary ovarian cancer cultures relative to that in healthy tube tissue cells (Figure 5).

#### Reversal of cisplatin resistance

**Figure 6:**
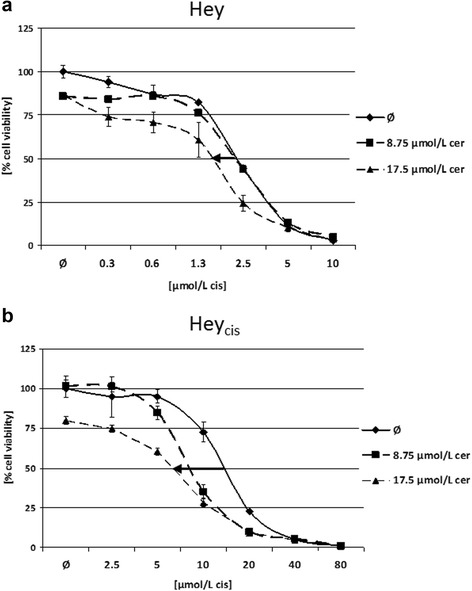
Effect of combined cerulenin/cisplatin treatment on cell viability. Effect of combined cerulenin/cisplatin treatment in the Hey **(a)** and cisplatin-resistant Hey_cis_ **(b)** cell lines. Cisplatin was given in various concentrations as a single agent or following administration of 8.75 μmol/L or 17.5 μmol/L cerulenin. As illustrated by the bold black arrows, sequential application of cerulenin for 6 h, followed by 72 h incubation in cisplatin-containing media shifted the cisplatin half maximum inhibitory concentration (IC50) from 2.3 μmol/L to 1.6 μmol/L (30% reduction; *P* = 0.004) in parental Hey cells and from 14.5 μmol/L to 6.6 μmol/L (54% reduction; *P* < 0.001) in the cisplatin-resistant Hey_cis_ cells.

Because of the generally greater FASN inhibition and anti-tumor effects achieved by cerulenin versus C75, experiments regarding cisplatin resistance were performed only with the former FASN inhibitor. Importantly, sequential but not simultaneous application of cerulenin and cisplatin reversed drug resistance. Cell line preincubation with 17.5 μmol/L cerulenin for 6 h followed by single-agent cisplatin application for an additonal 72 h, shifted the IC50 from 2.3 μmol/L to 1.6 μmol/L (30% reduction; *P* = 0.006) in Hey and from 14.5 μmol/L to 6.6 μmol/L (54% reduction; *P* < 0.001) in Hey_cis_ (Figure 6). Interestingly, 6 h of treatment with 8.75 μmol/L cerulenin already increased the cisplatin sensitivity in the cisplatin-resistant Hey_cis_ cells (41% IC50 reduction, from 14.5 nmol/L to 8.5 μmol/L; *P* = 0,002), whereas no effect was observed in the parental cells. (Identical treatment regime was applied to analyze reversal of cisplatin resistance in primary culture of 6 serous ovarian cancers compared to 2 primary cultures emanating from normal fallopian tube tissue and appropriate IC50 values were provided in Additional file 2: Table S2.)

#### Palmitic acid reverses the effect of FASN inhibition

**Figure 7:**
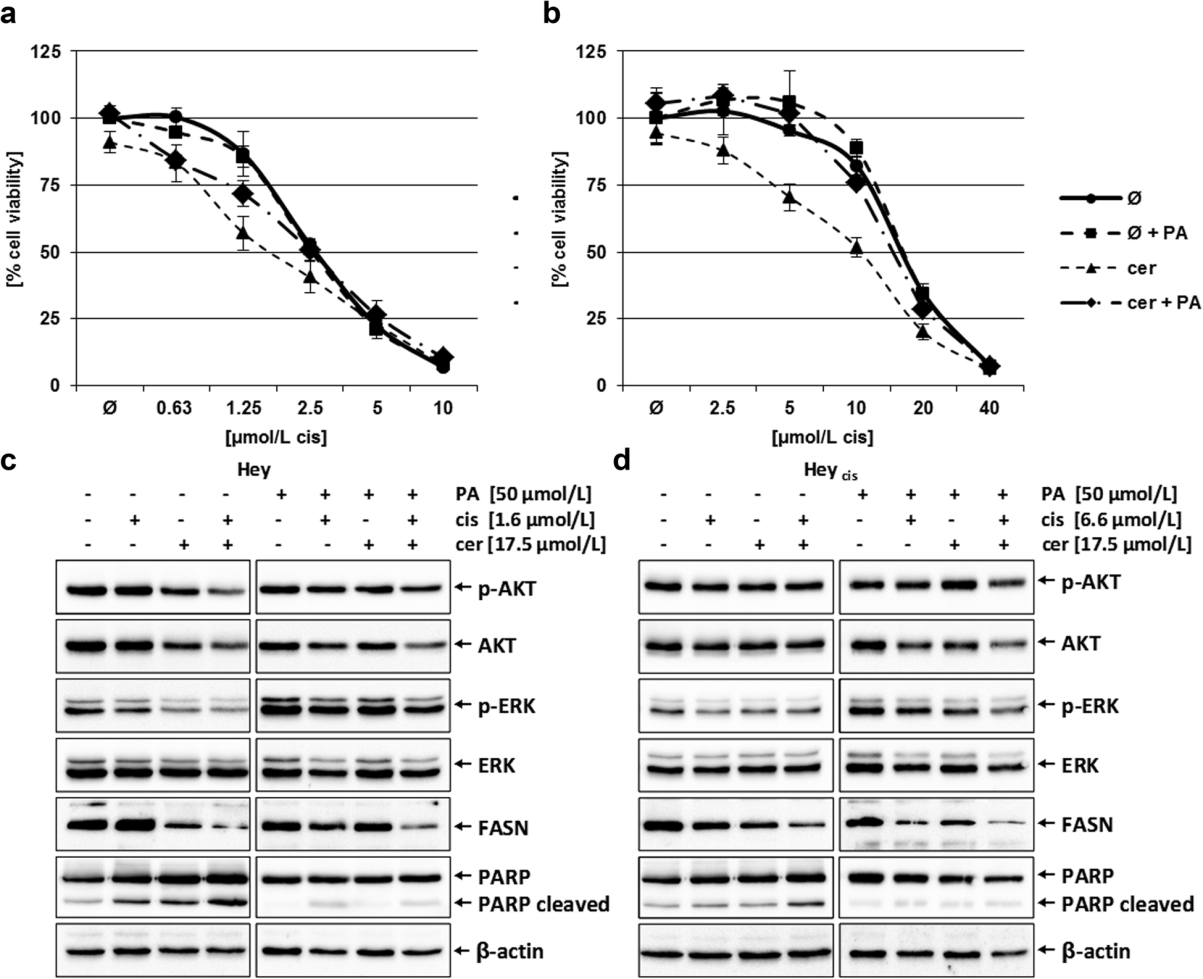
Effect of PA supplementation to combined cerulenin/cisplatin treatment in the Hey **(a, c)** and cisplatin-resistant Hey_cis_ **(b, d)** cells analyzing cell viability and proliferation and apoptosis. **(a**, **b)** Cisplatin was given in various concentrations as a single agent or following administration of 17.5 μmol/L cerulenin in the absence or presence of 50 μmol/L PA. Cell viability was investigated by the MTT assay. The shifted cisplatin IC50 from 2.7 μmol/L to 1.8 μmol/L (33% reduction; *P* = 0.007) in parental Hey cells after sequential application of cerulenin for 6 h, followed by 72 h incubation in cisplatin-containing media was completely reversed by administration of PA (both cisplatin IC50 of 2.6 μmol/L; *P* = 0.02) (a). In the Hey_cis_ cell line the shifted IC50 from 16.8 μmol/L to 10.5 μmol/L (38% reduction; *P* = 0.003) was partly abrogated by administration of PA (shifted cisplatin IC50 from 17.1 μmol/L to 15.5 μmol/L (only 9% reduction; *P* = 0.03) **(b)**. **(c**, **d)** Immunoblot analyses of phospho-AKT, AKT, phospho-ERK, ERK, FASN, PARP (total and cleaved) and β-actin (control) protein expression was investigated in in the Hey **(c)** and Hey_cis_ **(d)** cell lines after either single or combined cerulenin/cisplatin treatment in the absence or presence of PA with indicated concentrations. In the Hey cell line the slight dephosphorylation of AKT by combined treatment of cerulenin and cisplatin was less in the cells additionally treated with PA. In both the Hey and Hey_cis_ cell lines the reduction of the active pro-proliferative kinase phospho-ERK by drug treatment failed to appear in the additionally PA treated cells. Also, in both cell lines cerulenin diminished FASN protein expression in absence or presence of PA, but the significant induction of apoptosis (PARP cleavage) was prevented by adminstration of PA. (Quantitative analyses are provided in the Supplementary Materials – Additional file 2: Table S1.)

The Hey and Hey_cis_ cell lines were exposed to cerulenin (17.5 μmol/L) and various concentrations of cisplatin (0.63 – 40 μmol/L) in the presence or absence of exogeneous PA, the final product of FASN-catalyzed synthesis. As shown in Figure 7 the reduced cell viability after combined treatment of cerulenin and cisplatin is recovered by adding PA to the Hey cell line (7a; IC50 2.71 μmol/L to 1.78 μmol/L (*P* = 0.007) vs. 2.58 μmol/L to 2.55 μmol/L (*P* = 0.02)) as well as to the Hey_cis_ cell line (7b; 16.8 μmol/L to 10.5 μmol/L (*P* = 0.003) vs. 17.1 μmol/L to 15.5 μmol/L (*P* = 0.03)).

The immunoblot analyses showed that FASN protein expression is diminished by cerulenin independently on PA treatment, whereas the repression of the active pro-proliferative MAP kinase (phospho-ERK, p-ERK) and the induction of apoptosis (cleavage of PARP) exclusively occur in cells without PA administration (7c, d). The additional treatment with exogenous PA only induces minor repression of p-ERK and nearly no activation of apoptotic pathway (7c, d). The expression of the active form of AKT kinase (phospho-AKT) shows a slight repression by cerulenin after combined treatment with cisplatin. This is reversed by the additional treatment with PA in the Hey cell line (7c). (Quantitative analyses are provided in the Supplementary Materials – Additional file 2: Table S1.)

### ^18^ F-FCH uptake

In initial measurements, untreated Hey and Hey_cis_ cells showed markedly higher uptake of ^18^ F-FCH than of ^18^ F-FDG: respectively, Hey, 9.0% versus 1.2%, Hey_cis_, 3.5% versus 0.6% of administered activity. Subsequent experiments therefore were performed only with ^18^ F-FCH.

**Figure 8:**
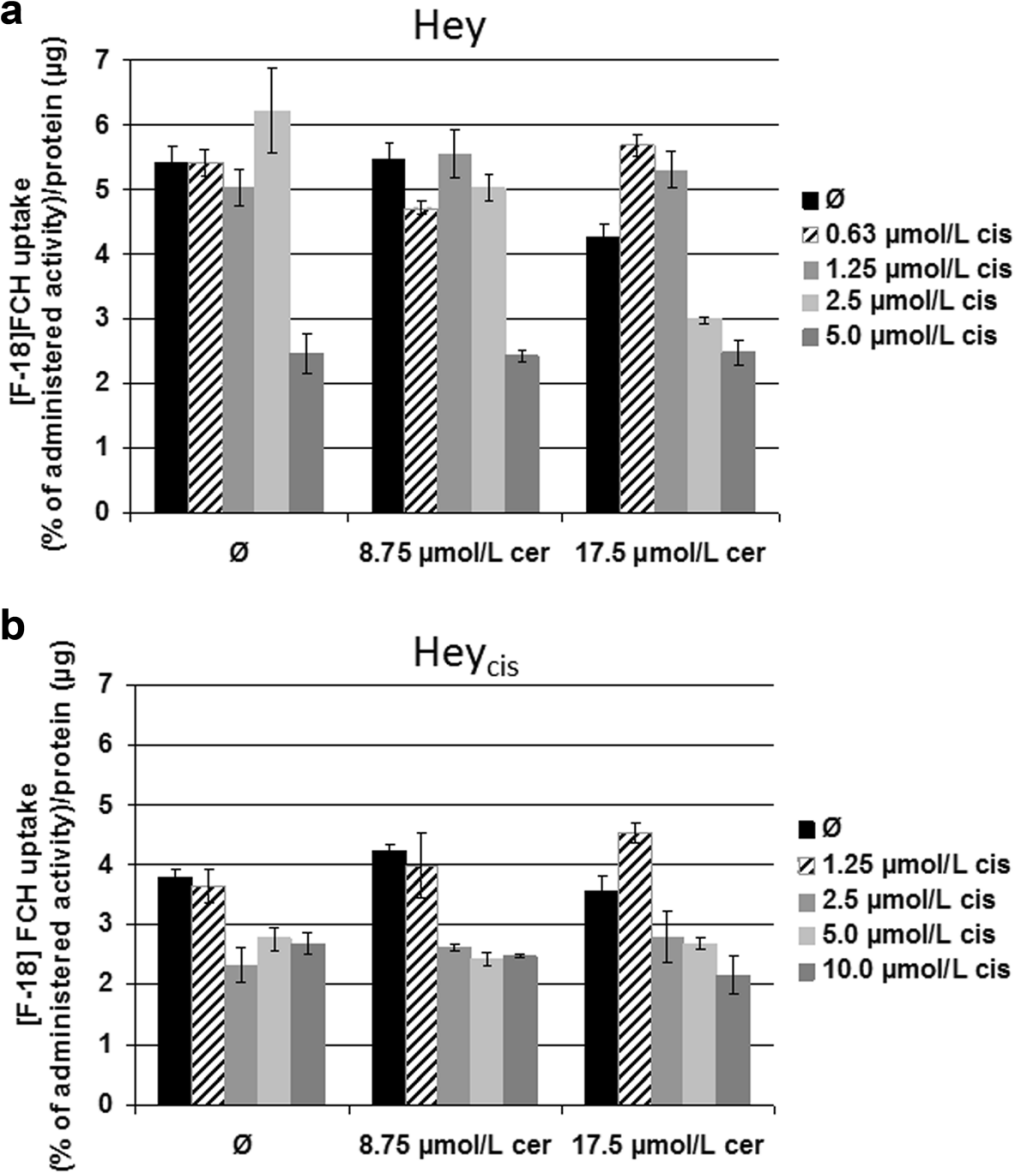
^18^ F-FCH uptake as a percentage of the administered activity normalized to protein content. ^18^ F-FCH uptake as a percentage of the administered activity normalized to protein content in the Hey **(a)** and cisplatin-resistant Hey_cis_ **(b)** cell lines. Cisplatin-resistant Hey_cis_ cells demonstrated lower mean ^18^ F-FCH uptake compared to parental Hey cells (5.42% ± 0.23% vs. 3.79% ± 0.14% of administered activity). At 2.5 μmol/L cisplatin, both tested cerulenin concentrations decreased ^18^ F-FCH uptake in cisplatin-resistant Hey_cis_ cells. For the parental Hey cells, this effect was only detectable at the higher cerulenin concentration. Treatment with cerulenin alone did not affect the cellular uptake of ^18^ F-FCH in any tested cell line. cis = cisplatin; cer = cerulenin.

Aligned with mRNA and protein expression results (Figure 2), and presumably reflecting their relatively lower FASN metabolic activity, the cisplatin-resistant Hey_cis_ cells demonstrated lower mean ^18^ F-FCH uptake compared to parental Hey cells: 5.42% ± 0.23% versus 3.79% ± 0.14% of administered activity (Figure 8).

Treatment with ≥2.5 μmol/L cisplatin concentrations induced changes in intracellular FCH uptake in both Hey and Hey_cis_ cell lines. Importantly, these effects were detectable only in cerulenin-pretreated cells. Moreover, corresponding to the MTT data, at a 2.5 μmol/L cisplatin concentration, both tested cerulenin concentrations (8.75 and 17.5 μmol/L) influenced the cisplatin tolerance and ^18^ F-FCH uptake in cisplatin-resistant Hey_cis_ cells (Figure 8). For the parental Hey cells, this effect was only detectable at the higher cerulenin concentration (17.5 μmol/L). The treatment with cerulenin alone did not affect the cellular uptake of ^18^ F-FCH in either tested cell line.

## Discussion

This *in vitro* investigation had three main findings. First, we confirmed and extended our earlier observations [7] and others [6,14,28] of considerable FASN overexpression in human ovarian cancer cells relative to that in healthy human tissue with a tendency towards higher expression in more aggressive, de-differentiated tumors. Cai et al. reported that the expression of FASN correlated with tumor grade and FIGO stage and is associated with HER2 expression in ovarian cancer; additionally, high expression of FASN was accompanied with poor prognosis of ovarian cancer [29]. In the present work, such overexpression was clearly demonstrated in immunohistochemical analysis of a TMA including tumors of high or low grades and different histological subtypes. Also, we showed marked FASN overexpression at the mRNA level by qRT-PCR and at the post-transcriptional level by WB in cell lines as well as primary tumor cultures of varying degrees of chemoresistance and differentiation. As stated in other studies for e.g. etoposide and paclitaxel [12,13], a correlation between multidrug resistance and FASN overexpression could not be proven in our experiments. In contrast, the drug sensitive Hey cell line demonstrated highest FASN expression level, whereas the single-drug resistant cell line Hey_cis_ and the multidrug resistent Skov-3 cell line displayed less FASN expression compared to the cisplatin-sensitive Hey cells.

Our second main finding was that in cell lines and primary cultures, the FASN inhibitor cerulenin strongly blocked FASN protein expression and both stimulated apoptosis and re-induced platinum sensitivity. Preclinical reversal of chemoresistance by FASN inhibition was described before in ovarian cancer and other human malignancies. Concomitant C75 and cisplatin administration augmented apoptosis in ovarian carcinoma cells compared to that seen with cisplatin alone [6]. A cerulenin/paclitaxel combination significantly inhibited growth in FASN-overexpressing, paclitaxel-resistant hepatocellular carcinoma cells [13]. In colon cancer low concentrations of a cerulenin/oxaliplatin combination enhanced apoptosis *in vitro* compared to the same concentrations of the drugs as single agents [30]. Additionally, the combination significantly inhibited tumor progression compared to that in untreated controls as well as in both single agent groups *in vivo* in xenotransplanted mice with severe combined immunodeficiency.

However, unlike other investigators [6,28,31], we detected a reducing effect of cerulenin on FASN protein expression and cell viability considerably exceeding that of C75 in ovarian cancer cell lines when the drugs were given as single agents. The only structural difference between these FASN inhibitors is the lack of the highly reactive epoxide moiety in C75 [4], presumably accounting for the lesser activity of C75.

Also in contrast to other work, we found that sequential application of a FASN inhibitor followed by the cytotoxic agent was more effective than was simultaneous application. The Uddin et al. study of C75 and cisplatin in ovarian cancer [6] and the Meena et al. study of cerulenin and paclitaxel in hepatocellular carcinoma [13], for example, administered both agents concomitantly or nearly so. Indeed, another study showed synergistic reduction of cell viability effects in breast cancer cell lines with concomitant C75/paclitaxel treatment, but antagonistic effects when C75 was given before paclitaxel [32]. We hypothesize that our observation that concomitant cerulenin/cisplatin treatment was less effective than sequential administration may be attributable to complexation of the two drugs when given simultaneously.

It was proposed that certain signaling pathways involved in cell apoptosis were closely associated with the inhibition of FASN, which helps to explain why FASN inhibitors may potentially be used to treat cancer. Other studies, however, have shown that palmitic acid, the final product of FASN pathway, is important for the formation of cell membranes [33], predominantly needed for the generation of phospholipids [34]. Therefore, the reduction of synthesized palmitic acid may be a reason to explain why the inhibition of FASN could induce apoptosis. The study by Zhao et al. showed a rescue effect of PA when additionally administered to quercetin, a FASN inhibitor that induced apoptosis in human liver cancer HepG2 cells [35]. In the current study, it was found that the observed effects of FASN inhibiton such as reduced cell viability, induction of apoptosis, and dephosphorylation of the pro-proliferative kinases ERK and AKT could be abrogate by adding exogenous PA.

Our third main finding was that ^18^ F-FCH showed potential utility as an ovarian cancer imaging agent. In both the Hey and Hey_cis_ lines, this radionuclide demonstrated considerably higher uptake than that of the clinically widely used ^18^ F-FDG. This observation, aligned with that of a clinical study showing that gynecologic tumors had higher ^11^C-choline than ^18^ F-FDG uptake [36], suggests potential for clinical use of ^18^ F-FCH in diagnostic positron emission tomography/computed tomography in ovarian cancer patients. Moreover, differential ^18^ F-FCH uptake by platinum-resistant versus platinum-non-resistant ovarian cancer cells raises the possibility of predicting platinum resistance *in vivo* by quantitative imaging. Such a biomarker-based strategy potentially could spare patients ineffective chemotherapy and resultant adverse events. Further studies in additional cell lines, animal models, and, if warranted, patients would be needed to establish clinical utility of ^18^ F-FCH in ovarian cancer. One potential issue that may need to be addressed is the possibility of intense physiological 18^F^-FCH uptake by the intestines obscuring smaller ovarian cancer lesions in the abdominal cavity.

Limitations of the present investigation should be considered. We could only include a small number of primary culture samples so far. Also, at this point we could only investigate one pair of platinum non-resistant parent/resistant daughter cell lines. Ideally, our findings should be confirmed in larger collections of patient materials as well as in xenograft models.

## Conclusions

Recently it has been shown, that adding the angiogenesis inhibitor bevacizumab to platinum/taxane combination regimens prolonged disease-free survival of advanced-stage ovarian cancer patients in the first-line setting [37-39]. Nonetheless, it remains important to develop additional treatments which, as single agents or combined with established cytostatic regimens, may overcome chemoresistance and improve survival of patients with this often lethal disease. Also in the management of recurrent/progressive disease, in which pegylated liposomal doxorubicin ± carboplatin is widely used, a marginal progression free survival advantage was observed only in platinum-sensitive setting and second-line treatment [40]. Therefore, the present results regarding FASN overexpression and FASN inhibitor cytotoxic effects in chemosensitive, but also in platinum-resistant ovarian cancer cells make an important contribution and may have an impact on treatment in the foreseeable future.

Cerulenin is unapproved for use in humans. However, other FASN inhibitors such as orlistat, which has been shown to reduce FASN activity in non-small cell lung cancer [41], are readily available for clinical use, albeit issues of that drug’s low cell permeability and solubility, lack of selectivity [42], and poor metabolic stability [43] may need to be overcome, perhaps through use of orlistat metabolites.

Further research in animal models is warranted to assess whether FASN inhibition can overcome platinum resistance and improve survival. Study also should be undertaken regarding FASN inhibition to reverse chemoresistance to other drug classes such as taxanes.

## Additional files

Additional file 1:
**Fatty Acid Synthase Overexpression: Target for Therapy and Reversal of Chemoresistance in Ovarian Cancer.** Description of additional materials and methods Immunhistochemical analyses, ovarian cancer cell lines and primary cell culture material, RNA isolation, reverse transcription and quantitative real-time polymerase chain reaction (qRT-PCR), Western Blot (WB), 3- (4,5-dimethylthiazol-2-yl)- 2,5-diphenyltetrazolium-bromide (MTT) assay, cell death detection enzyme-linked immunoassay (CDDE). Additional file 2: Table S1. Quantification of protein expression by the analyzing software Image J®. Quantification of protein expression of phospho-AKT, AKT, phospho-ERK and ERK in cis, cer, and PA-treated Hey and Hey_cis_ cells vs. untreated cells normalized to β-actin by the analyzing software Image J®. **Table S2.** IC50 values of cisplatin in primary culture combined with cerulenin treatment. Cell viability test (MTT assay) in primary cultures after combined treatment of cerulenin and cisplatin. The table shows the appropriate IC-50 values of cisplatin in 6 primary cultures of serous ovarian cancer, G3, with various resistances against cisplatin and 2 primary culture of healthy fallopian tube tissue after single treatment, combined treatment with 8.75 μmol/L cerulenin, or combined treatment with 17.5 μmol/L cerulenin.

## Abbreviations

FASN: Fatty acid synthase
PA: Palmitic acid
IC50: Half maximum inhibitory concentration
