# Supplementary material for: Fatty acid synthase overexpression: target for therapy and reversal of chemoresistance in ovarian cancer
Source: J Transl Med. 2015 May 7;13:146. doi: 10.1186/s12967-015-0511-3 (PMC4504229; doi:10.1186/s12967-015-0511-3)
Supplement: Additional file 1: — Fatty Acid Synthase Overexpression: Target for Therapy and Reversal of Chemoresistance in Ovarian Cancer. Description of additional materials and methods Immunhistochemical analyses, ovarian cancer cell lines and primary cell culture material, RNA isolation, reverse transcription and quantitative real-time polymerase chain reaction (qRT-PCR), Western Blot (WB), 3- (4,5-dimethylthiazol-2-yl)- 2,5-diphenyltetrazolium-bromide (MTT) assay, cell death detection enzyme-linked immunoassay (CDDE). [file 12967_2015_511_MOESM1_ESM.pdf]

ORIGINAL RESEARCH PAPER

Fatty Acid Synthase Overexpression: Target for Therapy and Reversal of Chemoresistance in Ovarian Cancer. – ADDITIONAL MATERIAL

Dirk O. Bauerschlag[1]*, Nicolai Maass[1]*, Peter Leonhardt[1], Frederik A. Verburg[2], Ulrich Pecks[1], Felix Zeppernick[1], Agnieszka Morgenroth[2], Felix M. Mottaghy[2], Rene Tolba[3], Ivo Meinhold-Heerlein[1+], and Karen Bräutigam[1+]

Departments of [1]Gynecology and Obstetrics or [2]Nuclear Medicine, [3]Institute for Laboratory Animal Science, University Medical Center RWTH Aachen, Pauwelsstrasse 30, 52074 Aachen, Germany

Running title: FASN overexpression: target in ovarian cancer

Corresponding author:

Dr. rer. nat. Karen Bräutigam

Molecular Gynecology, Department of Gynecology and Obstetrics

University Medical Centre RWTH Aachen

Pauwelsstrasse 30,

52074 Aachen, Germany

phone +49/241/8037051

fax +49/241/8082476

e-mail: kbraeutigam@ukaachen.de

*These authors contributed equally, and [+]these authors share senior authorship of this work.

ADDITIONAL MATERIALS AND METHODS

Immunhistochemical analyses. Tissue microarray (TMA) slides were manually processed and stained for fatty acid synthase (FASN) expression levels. The streptavidin-biotin peroxidase technique with diaminobenzidine as chromogen was applied. Slides were placed in citrate buffer pH 9.0 and placed in a boiling water bath at 95°C for 1 h. Endogenous peroxidase activity was suppressed using 5% hydrogen peroxidase solution in methanol. For each slide, 150 µL of anti-FASN antibody (ab99359, Abcam, Cambridge, UK) was applied in a 1:200 dilution, followed by horseradish peroxidase [HRP]-conjugated anti-rabbit IgG (NA934, GE Healthcare, Waukesha, WI, USA) in a 1:100 dilution. TMA slides omitting the anti-FASN antibody served as negative controls. All slides were counterstained with hematoxylin (Real Hematoxylin 1:3, Dako, Glostrup, Denmark), dehydrated, cleared, and cover-slipped with premount. A NanoZoomer Digital Pathology System (Hamamatsu Photonics, Hamamatsu, Japan) was used to determine the immunoreactive score (IRS), which incorporates protein staining intensity and the percentage of protein-positive cells.[1]

Ovarian cancer cell lines and primary cell culture material

Growing cells were split at 80% confluency. Cell numbers were determined via live counting with trypan blue using a Neubauer chamber and plated in appropriate number in 96-well plates or cell culture dishes according to the planned experiment.

RNA isolation, reverse transcription and quantitative real-time polymerase chain reaction (qRT-PCR). $4x10^5$ cells were plated in 1 mL RPMI medium in each well of a 6-well plate. After overnight growth, RNA was

extracted using an Rneasy kit (Qiagen, Hilden, Germany) per supplier recommendations. After photometric quantification of each sample's RNA, 1 µg was reverse transcribed into cDNA using an iScript kit (Bio-rad Laboratories, Inc., Hercules, CA, USA), per manufacturer recommendations. qRT-PCR was performed in a StepOne cycler (Applied Biosystems, Foster City, CA, USA) using 2XSYBR Green Supermix (Bio-Rad) according to manufacturer recommendations. In brief, reactions consisted of 1 µL forward primer (0.5 µmol/L), 1 µL reverse primer (0.5 µmol/L), 10 µL SYBR Green Supermix (1X) and 1 µL cDNA (1/20 of the reverse transcription approach) as template. Beta-actin was used as expression control with subsequent primer sequences: FASN F1 5' TAT GCT TCT TCG TGC AGC AGT 3'; FASN R1 5' GTG GAT GAT GCT GAT GAT GGA 3'; Actin F1 5' TCG TGC GTG ACA TTA AGG AG 3'; Actin R1 5' GTC AGG CAG CTC GTA GCT CT 3'.

Overall, PCR reactions had a 20 µL volume, adjusted with nuclease-free water. PCR product specificity was verified by comparative melting curve analysis. Cycle threshold values were quantified, and normalized to beta-actin expression, and relative expression was calculated. The cycler program comprised an initial denaturation step at 95°C for 3 min, followed by 40 amplification cycles, each including a denaturation step (15 s at 95°C), an annealing step (15 s at 54°C), and an elongation step (30 s at 72°C).

Western Blot (WB). $4x10^5$ cells were plated in 1 mL RPMI medium in each well of a 6-well plate. After overnight growth, either the reagent to be tested was added at the desired concentration or the cells were left untreated. For protein isolation, cells were washed with cold phosphate-buffered saline (PBS), scraped off culture dishes, and collected in 1500 µL ice-cold PBS.

After centrifugation at 4°C at 4,000 rpm for 5 min, supernatant was removed and the pellet resuspended in 80-100 µL denaturing lysis buffer (1% sodium dodecyl sulfate [SDS], 1 mmol/L sodium orthovanadate, 10 mmol/L Tris pH 7.4, 2% protease inhibitor). After incubation at 95°C for 5 min, the sample was vortexed and centrifuged at 4°C and 13,200 rpms for 15 min. After protein concentration determination by DC Protein Assay (Bio-Rad) per manufacturer recommendations, protein samples were diluted to desired concentrations with PBS and 5x loading buffer. Following boiling at 95°C for 5 min, protein samples were pipetted into prepared SDS gels composed of a collection layer and a separation layer (respectively 3% and 7.5% – 12% acrylamide). Electrophoresis was conducted using the Mini-PROTEAN Tetra Cell (Bio-Rad) at 300 V/15 mA for 30 min, then at 300 V/30 mA for each gel, to the minimum required protein size. Protein transfer onto the Hybond-P polyvinylidene difluoride membrane (GE Healthcare) was carried out in a Trans-blot SD system (Bio-Rad) at a constant 25 V for 60 min. Afterwards, membranes were blocked at room temperature in 5% bovine serum albumin-Tween20-tris-buffered saline (BSA-TTBS) solution for 1 h to avoid non-specific membrane binding before primary antibody application. Per manufacturer guidelines, ab99359 (Abcam) and beta-actin (A5441, Sigma-Aldrich Corporation, St. Louis, MO, USA), respectively diluted to 1:500 or 1:4,000 in 5% BSA-TTBS, were incubated overnight at 4°C with the membrane rotating. The corresponding secondary antibodies, HRP-conjugated anti-rabbit IgG (NA934, GE Healthcare) or HRP-conjugated anti-mouse IgG (NA931, GE Healthcare), each diluted 1:2000 in 5% BSA-TTBS, were added to the membrane and agitated at room temperature for 2 h. For

detection, membranes were overlaid with 2 mL of ECL WESTZOL solution (iNtRON Biotechnology, Gyeonggi-do, South Korea) for 1 min and developed using a chemiluminescence imager (JENOPTIK AG, Jena, Germany). Before membranes were blocked again and incubated with new primary antibodies, previously-applied antibodies were removed using WB stripping buffer at 55°C for 60 min.

3- (4,5-dimethylthiazol-2-yl)- 2,5-diphenyltetrazolium-bromide (MTT) assay. The glycolysis rate of viable cells was determined by MTT assay. For this purpose, $4x10^3$ cells were plated in 50 µL RPMI medium in each well of a 96-well plate. After overnight growth, the reagent to be tested was added at the desired concentration. For final detection, 10 µL MTT Stock Solution (5 mg/mL) per 100 µL medium was applied 72 h (ovarian cancer cell lines) and 96 h (primary cultures) after drug treatment. After incubation for 4 h at 37°C, formazan crystal formation was stopped with 150 µL solubilisation solution (0.04 N HCl in 100% isopropanol) per 10 µL MTT solution, and the crystals were resolved by consistent shaking at room temperature for 15 min. The absorption coefficient was determined at a 590-nm wavelength, with a 630-nm reference wavelength, using an EL800 microplate reader (BioTek, Winooski, VT, USA).

Cell death detection enzyme-linked immunoassay (CDDE). The Cell Death Detection ELISA^{PLUS} (Roche Diagnostics, Mannheim, Germany) was used per manufacturer specifications; $4x10^3$ cells/well were seeded in 96-well plates and allowed to attach overnight. After 25 µmol/L cerulenin treatment for 24 h (time frame according to manufacturer's recommendation resulting in optimal detection of apoptosis) and subsequent cell lysis as recommended by

manufacturer, apoptosis was measured using the EL800 microplate reader at a 405-nm wavelength, with a 490-nm reference wavelength.

ADDITIONAL REFERENCES

1. Remmele W, Stegner HE (1987) [Recommendation for uniform definition of an immunoreactive score (IRS) for immunohistochemical estrogen receptor detection (ER-ICA) in breast cancer tissue]. *Pathologe* **8**(3)**:** 138-40
