# Supplementary material for: Fatty acid synthase overexpression: target for therapy and reversal of chemoresistance in ovarian cancer
Source: J Transl Med. 2015 May 7;13:146. doi: 10.1186/s12967-015-0511-3 (PMC4504229; doi:10.1186/s12967-015-0511-3)
Supplement: Additional file 2: Table S1. — Quantification of protein expression by the analyzing software Image J®. Quantification of protein expression of phospho-AKT, AKT, phospho-ERK and ERK in cis, cer, and PA-treated Hey and Heycis cells vs. untreated cells normalized to β-actin by the analyzing software Image J®. Table S2. IC50 values of cisplatin in primary culture combined with cerulenin treatment. Cell viability test (MTT assay) in primary cultures after combined treatment of cerulenin and cisplatin. The table shows the appropriate IC-50 values of cisplatin in 6 primary cultures of serous ovarian cancer, G3, with various resistances against cisplatin and 2 primary culture of healthy fallopian tube tissue after single treatment, combined treatment with 8.75 μmol/L cerulenin, or combined treatment with 17.5 μmol/L cerulenin. [file 12967_2015_511_MOESM2_ESM.pdf]

A2 Table S1. Quantification of protein expression by the analyzing software Image J®

Hey					Hey			
	Ø	cis	cer	cis+cer	Ø + PA	cis + PA	cer + PA	cis+cer + PA
p-AKT	1.00	0.89	0.79	0.54	1.00	1.17	0.96	1.00
AKT	1.00	0.82	0.67	0.58	1.00	1.00	0.96	0.74
p-ERK	1.00	0.49	0.30	0.26	1.00	1.06	0.95	0.87
ERK	1.00	0.81	0.86	0.95	1.00	1.00	1.04	1.05

Hey cis					Hey cis			
	Ø	cis	cer	cis+cer	Ø + PA	cis + PA	cer + PA	cis+cer + PA
p-AKT	1.00	0.77	0.73	0.71	1.00	1.19	1.32	1.31
AKT	1.00	0.78	0.75	0.67	1.00	0.80	0.82	0.82
p-ERK	1.00	0.57	0.64	0.62	1.00	0.98	0.80	0.94
ERK	1.00	0.84	0.87	0.79	1.00	0.89	0.93	0.93

A2 Table S2. IC50 values of cisplatin in primary culture combined with cerulenin treatment

	**Ø**	**+ 8.75 µmol/L cer (% reduction)**	**+ 17.5 µmol/L cer (% reduction)**
**G3#1**	4.11 µmol/L cis	4.05 µmol/L cis*** (1.5%)	1.61 µmol/L cis*** (60.8%)
**G3#2**	24.50 µmol/L cis	14.44 µmol/L cis* (41.1%)	8.51 µmol/L cis** (65.3%)
**G3#3**	37.51 µmol/L cis	24.32 µmol/L cis* (35.2%)	5.24 µmol/L cis*** (86.0%)
**G3#4**	9.86 µmol/L cis	7.02 µmol/L cis** (28.8%)	0.82 µmol/L cis** (91.7%)
**G3#5**	30.32 µmol/L cis	29.61 µmol/L cis*** (2.3%)	17.01 µmol/L cis*** (43.9%)
**G3#6**	3.76 µmol/L cis	4.14 µmol/L cis* (-)	1.65 µmol/L cis** (56.1%)
**C1**	13.54 µmol/L cis	15.05 µmol/L cis** (-)	9.19 µmol/L cis** (32.1%)
**C2**	34.33 µmol/L cis	32.96 µmol/L cis** (4.0%)	29.63 µmol/L cis* (13.7%)

(*** $P < 0.001$; ** $P < 0.01$; * $P < 0.05$)
